# Lithium Augmentation in Treatment‐Resistant Depression: A Qualitative Review of the Literature

**DOI:** 10.1002/phar.70063

**Published:** 2025-09-23

**Authors:** Angela Acero‐González, Yahira Guzman, Nadia Juliana Proaños, Rosa‐Helena Bustos, María Aconcha, Ivan Guerrero, Laura Alejandra Martinez, Michael Berk, Seetal Dodd

**Affiliations:** ^1^ Grupo de Investigación en Psiquiatría y Salud Mental, Departamento de Salud Mental, Facultad de Medicina Universidad de La Sabana y Clínica Universidad de La Sabana Chía Colombia; ^2^ Grupo de Investigación en Salud, Departamento de Epidemiología, Facultad de Medicina Universidad de La Sabana y Clínica Universidad de La Sabana Chía Colombia; ^3^ Department of Clinical Pharmacology, Evidence‐Based Therapeutics Group, Faculty of Medicine Universidad de La Sabana and Clínica Universidad de La Sabana Chía Cundinamarca Colombia; ^4^ Faculty of Medicine Universidad de La Sabana Chía Colombia; ^5^ IMPACT ‐ the Institute for Mental and Physical Health and Clinical Translation, School of Medicine Deakin University, Barwon Health Geelong Australia; ^6^ Centre for Youth Mental Health University of Melbourne Parkville Australia

**Keywords:** augmentation therapy, depression, lithium, mental health, mood disorders, psychiatry, treatment‐resistant‐depression

## Abstract

Depression is the leading cause of disability worldwide, affecting people of all ages. Both pharmacological and non‐pharmacological therapies are available for its treatment. However, some patients do not respond to first‐line pharmacological interventions, referred to as treatment‐resistant depression (TRD). Individuals with TRD face a significantly higher risk of mortality, including an increased risk of suicide. Additionally, TRD poses a substantial economic burden on health care systems. Various treatment options have been explored for TRD, including augmentation of an antidepressant through the use of an additional agent. Lithium salts have shown promising benefits in the TRD. Lithium requires close therapeutic monitoring due to its narrow therapeutic range, with well‐defined thresholds for efficacy and toxicity, in addition to its pharmacokinetic characteristics. Furthermore, lithium has been associated with a reduced risk of mortality by lowering aggression, impulsivity, and suicide rates. Compared with other agents used in the management of TRD—such as atypical antidepressants, second‐generation antipsychotics (SGAs), ketamine, and thyroid hormones—lithium is considered a cost‐effective augmentation option, alongside other evidence‐based strategies, and has a well‐established efficacy profile. This literature review examines the role of lithium as an augmentation agent in TRD, with a focus on its pharmacological and clinical properties, as well as the current evidence supporting its use.

## 1 Introduction

Depression is a major contributor to the global disease burden [1]. It is estimated that 350 million people worldwide suffer from this condition [2]. Depression is characterized by a persistent alteration in mood that affects multiple domains of mental functioning: sleep, attention, motivation, cognitive and motor patterns, eating behavior, energy levels, and so forth, which impair an individual's functioning and increase morbidity and mortality risks [3]. Within this group, around 100 million people are estimated to suffer from treatment‐resistant depression (TRD), which is associated with even greater disability, impaired quality of life, and economic and social impacts [4].

The causes of depression are incompletely understood; however, there is evidence that depression is a complex interaction of biological, genetic, psychosocial, and environmental factors [5]. Antidepressant medications and cognitive behavioral therapy (CBT) are the main treatments for major depressive disorder (MDD), but 30%–50% of patients do not respond sufficiently to first‐line treatment [6].

Patients with treatment‐resistant depression have a higher risk of mortality, including due to suicide. However, mortality in patients with treatment‐resistant depression is also increased by other medical conditions or unknown causes. This underscores the need to prevent these complications through timely treatment of major depressive disorders and, eventually, treatment‐resistant depression [7]. The years of life lost and the direct and indirect costs associated with the treatment of MDD and TRD have a negative impact on the health and well‐being of the affected population. These effects include educational aspects, relationship stability, fertility, quality of childcare, and family income, among others [8].

There is no single definition of TRD; however, many studies adopt the definition used by the United States Food and Drug Administration (FDA) and the European Medicines Agency (EMA), which requires a minimum of two prior treatment failures and confirmation of adequate dosage and treatment duration (4–6 weeks) [9]. Other proposals stratify TRD based on multiple variables, such as the number and type of treatments used, the number of treatments, the duration of the depressive episode, and the severity of depression, as well as functional, personality, and contextual factors, among others [10]. This highlights the complexity of TRD, in which multiple risk factors, such as early‐life trauma, symptom severity, episode duration, psychotic symptoms, cognitive impairments, and comorbid disorders including personality and anxiety symptoms, play a role [4].

Several pharmacotherapeutic options to treat TRD have been studied, including increasing the dose of ongoing antidepressants, switching to another antidepressant, combining antidepressants, or augmentation with another medication with antidepressant effects.

However, evidence shows that augmentation with a second non‐antidepressant drug, such as lithium, second‐generation antipsychotics (SGA), thyroid hormones, ketamine, and dopaminergic agents, is the most effective option for TRD, with quetiapine and lithium being the most studied and well known [11].

## 2 Lithium Carbonate (Li_2_CO_3_) as a Therapeutic Agent

Lithium has been the cornerstone of treatment for bipolar disorder, for acute manic episodes, and long‐term prophylaxis to prevent new illness episodes [12]. Though lithium has shown similar efficacies in acute manic episodes when compared to other mood stabilizers, including valproate and SGA, such as olanzapine and haloperidol [13], its prophylactic properties have been demonstrated. An observational study comparing monotherapy with lithium and monotherapy with other mood stabilizers found that prophylactic lithium monotherapy was more effective as maintenance therapy than valproate, lamotrigine, olanzapine, and quetiapine [14]. In addition, lithium can reduce mortality by reducing the risk of suicide. The antisuicidal effect of lithium was shown in several meta‐analyses [15, 16], including one study that found that the risk of committing suicide was five times lower among patients with bipolar disorder, schizoaffective disorder, or major depressive disorder who received lithium therapy compared to patients who received other treatments (e.g., antidepressants, anticonvulsants, antipsychotics, or placebo) [15].

Lithium carbonate (Li_2_CO_3_) is an inorganic salt widely used in medical practice (Table 1). It has been employed as a treatment for mood‐related psychiatric disorders, earning recognition as a mood‐stabilizing agent since 1949 [17, 18]. Lithium has a narrow therapeutic index, necessitating careful monitoring to prevent adverse effects [19]. Lithium orotate, a dietary supplement available without a prescription, has reemerged as a potential therapeutic option for bipolar disorder and depression. However, there is insufficient evidence to confirm its effectiveness or establish clinical guidelines for its use, and it is not approved by the FDA [20].

**TABLE 1:** Physicochemical and chemical properties of lithium carbonate (Li_2_CO_3_).

Structure 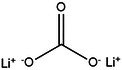	
Chemical formula	Li_2_O_3_
Synonyms	Lithonate, Lithium carbonate, Lithi carbonas, Dilithium carbonate
Chemical structure	Composed of two lithium cations (Li^+^) and one carbonate anion (CO_3_^2−^). The carbonate ion consists of one carbon atom covalently bonded to three oxygen atoms, with a trigonal planar geometry
Appearance	White, odorless crystalline powder
Molecular weight (amu)	73.89
Melting point	723°C (1333°F)
Boiling point	Decomposes before boiling; forms lithium oxide (Li_2_O) and carbon dioxide (CO_2_) at high temperatures
Solubility in water	Slightly soluble; 13.3 g/L at 20°C, decreasing with increasing temperature. Solubility is pH‐dependent due to the carbonate ion's basicity
pH in Aqueous solution	Approximately 11–12 (basic due to hydrolysis of the carbonate ion). The basic pH of lithium carbonate solutions results from the partial hydrolysis of the carbonate ion (CO_3_^2−^ + H_2_O ⇌ HCO_3_^−^ + OH^−^)

### 2.1 Pharmacodynamic

The mechanism of action of lithium remains incompletely understood, although various theories have been proposed. One of the most extensively described mechanisms is the inhibition of glycogen synthase kinase‐3 (GSK‐3), an enzyme that plays a critical role in regulating numerous cellular functions, including dopaminergic and glutamatergic neurotransmission, synaptic plasticity, inflammation, circadian rhythm regulation, and apoptosis. Lithium‐induced inhibition of GSK‐3 may contribute to its antidepressant effects by modulating these pathways, enhancing neuronal plasticity, and promoting neuronal survival [21].

Additionally, lithium promotes the expression of brain‐derived neurotrophic factor (BDNF), a key factor for neurogenesis and neuronal repair [22]. BDNF acts on the mesocortical dopaminergic circuit, which may mediate its antidepressant effects. Evidence from animal models demonstrates that this mechanism involves the activation of dopaminergic neurons in the ventral tegmental area projecting to the medial prefrontal cortex [23].

Furthermore, lithium modulates intracellular signaling by displacing cations such as sodium and magnesium at specific binding sites, which can alter the function of key proteins involved in neuronal signal transmission. This displacement may stabilize inactive conformations of certain receptors, thereby reducing hyperactive signaling associated with mood disorders [24]. Lithium has extensive effects on intracellular calcium second messenger signaling.

Lithium also modulates neurotransmitters by inhibiting excitatory neurotransmission mediated by dopamine and glutamate while enhancing inhibitory neurotransmission mediated by gamma‐aminobutyric acid (GABA). Regarding serotonin, some studies suggest that lithium potentiates serotonergic neurotransmission, contributing to its antidepressant effect. This potentiation is particularly observed when lithium is combined with tricyclic antidepressants (TCAs) [25].

**FIGURE 1:**
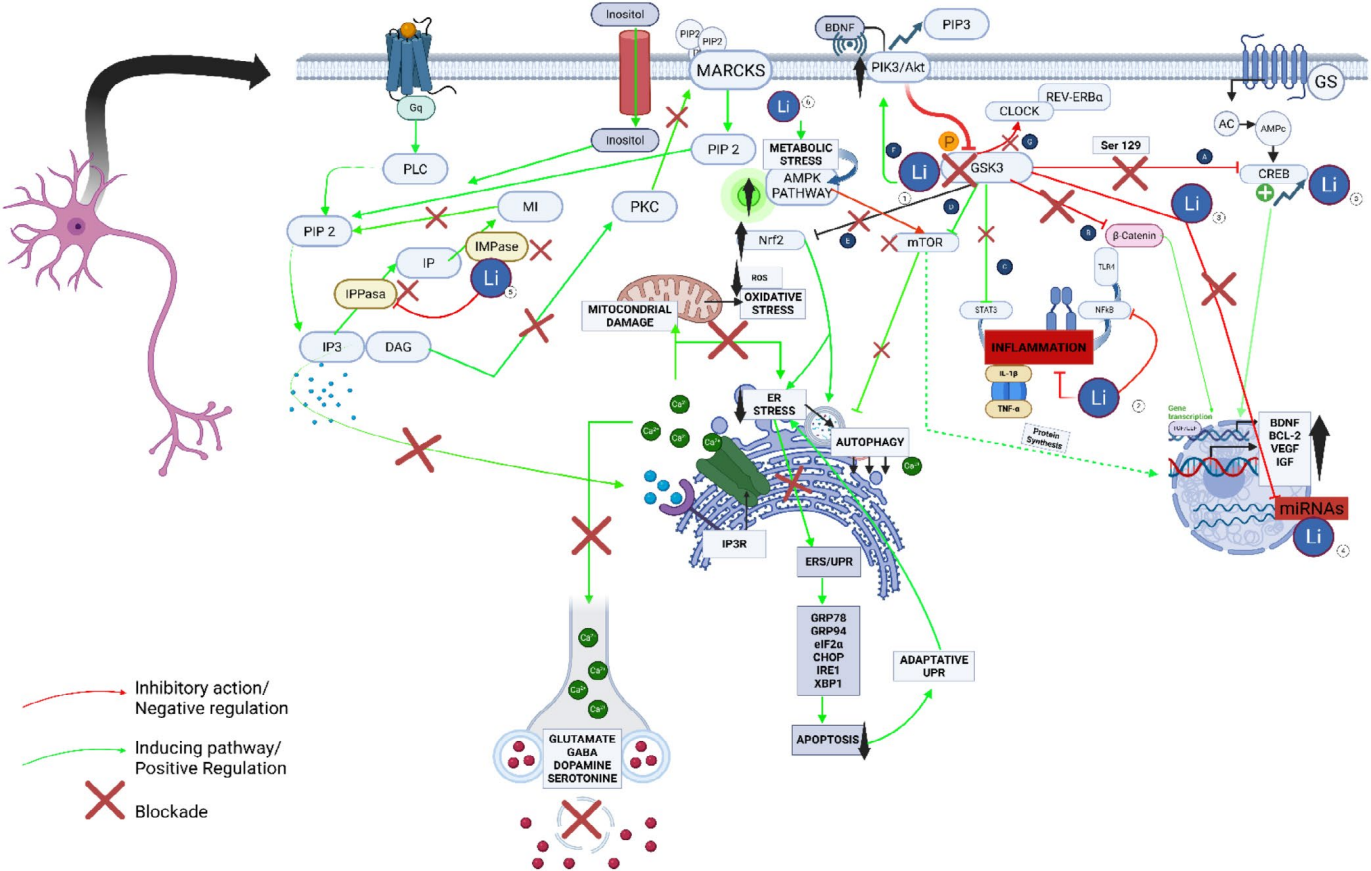
Representative schematic of the main mechanisms of action of lithium (Li+), including its specific molecular targets and the pathways it affects [27, 28, 29, 30, 31]. Lithium (Li^+^) exerts five principal mechanisms across distinct molecular pathways: (1) it inhibits GSK3, thereby disinhibiting the CREB pathway and potentiating Wnt/β‐catenin, which promotes expression of neuroprotective factors (BDNF, BCL‐2), while simultaneously suppressing pro‐inflammatory signals mediated by NFκB and STAT3, activating Nrf2 and modulating autophagy, stimulating the PI3K/Akt complex to further reinforce GSK3 inhibition, and activating the clock proteins CLOCK and REV‐ERBα to regulate circadian rhythms; (2) it regulates mTOR and AMPK—initially activating mTOR following GSK3 inhibition but, under metabolic stress, triggering AMPK to inhibit mTOR and thereby promote nuclear protein synthesis and selective endoplasmic reticulum autophagy; (3) it acts complementarily on NFκB by directly inhibiting this pro‐inflammatory complex; (4) it further enhances the CREB pathway and miRNA‐mediated regulatory mechanisms to boost transcription of neuroprotective factors; and (5) it blocks the IPPase and IMPase enzymes, reducing generation of IP_3_ and DAG, which diminishes PKC and MARCKS activation, retains PIP_2_ in the membrane, reduces Ca^2+^ release from the endoplasmic reticulum, and dampens synaptic neurotransmitter release—collectively contributing to decreased ER stress, protein misfolding, apoptosis, and inflammation. AC, adenylate cyclase; Akt, protein kinase B; AMPK, AMP‐activated protein kinase; BCL‐2, B‐cell lymphoma 2; BDNF, brain‐derived neurotrophic factor; Ca^2+^, calcium; cAMP, cyclic adenosine monophosphate; CHOP, C/EBP homologous protein; CLOCK, Circadian Locomotor Output Cycles Kaput; CREB, cAMP response element‐binding protein; DAG, diacylglycerol; eIF2α, eukaryotic initiation factor 2 alpha; ERS/UPR, endoplasmic reticulum stress/unfolded protein response; G, G protein–coupled receptor; GRP78, glucose‐regulated protein 78; GRP94, glucose‐regulated protein 94; GSK3, glycogen synthase kinase‐3; IFNγ, interferon gamma; IGF, insulin‐like growth factor; IL‐1β, interleukin‐1 beta; IMPase, inositol monophosphatase; IP, inositol monophosphate; IP₂, inositol 1,4‐bisphosphate; IP₃, inositol 1,4,5‐trisphosphate; IP₃R, inositol 1,4,5‐trisphosphate receptor; IPPase, inositol polyphosphate phosphatase; IRE1, inositol‐requiring enzyme 1; Li^+^, lithium ion; MARCKS, myristoylated alanine‐rich C‐kinase substrate; MI, myo‐inositol; miRNAs, microRNAs; mTOR, mechanistic target of rapamycin; NFκB, nuclear factor kappa B; Nrf2, nuclear factor erythroid 2‐related factor 2; PI3K, phosphatidylinositol 3‐kinase; PIP₂, phosphatidylinositol 4,5‐bisphosphate; PIP₃, phosphatidylinositol 3,4,5‐trisphosphate; PKC, protein kinase C; PLC, phospholipase C; REV‐ERBα, nuclear receptor subfamily 1 group D member 1; ROS, reactive oxygen species; STAT3, signal transducer and activator of transcription 3; TCF/LEF, T‐cell factor/lymphoid enhancer factor; TLR4, Toll‐like receptor 4; TNF‐α, tumor necrosis factor alpha; VEGF, vascular endothelial growth factor; Wnt/β‐cat, Wnt/β‐catenin pathway; XBP1, X‐box binding protein 1.

Finally, lithium exhibits immunomodulatory effects, suggesting that its ability to modify immune responses may be part of its mechanism of action in mood disorders and has effects on mitochondrial energy generation [26, 27, 28, 29, 30, 31] (Figure 1).

### 2.2 Pharmacokinetics

Lithium primarily follows linear or first‐order kinetics, meaning its elimination is proportional to its plasma concentration. As the dose increases, lithium plasma concentrations rise in a quasi‐linear manner [32]. Regarding lithium pharmacokinetics, the following parameters describe the processes by which the ion is released, absorbed, distributed, metabolized, and eliminated. This drug transformation process is recognized by the acronym LADME, which stands for liberation, absorption, distribution, metabolism, and excretion. In this model, liberation refers to the release of the active pharmaceutical ingredient from its dosage form prior to absorption. Lithium is administered orally in the form of lithium carbonate, available in immediate‐ or extended‐release capsules, tablets, and oral solution (Table 2) [37].

**TABLE 2:** Lithium pharmacokinetics and therapeutic drug monitoring [33, 34, 35, 36].

PK parameter	Lithium
*C*_max_, mmol/L	0.5–1.2 mmol/L dose dependent
*T*_max_, hours	1–3 h (immediate release)
*Distribution*: Widely distributed throughout total body water	
Protein binding, %	Lithium crosses cell membranes slowly and does not bind significantly to plasma proteins. < 10% (Low plasma protein binding)
Vd/F, L/kg, range	Approximately 0.7–1.0 L/kg
Metabolism	N/A. Lithium does not suffer any significant liver metabolic transformation. Lithium is excreted unchanged by the kidneys
*Elimination*: Primarily renal excretion (> 95% of the dose). Lithium is 100% filtered by the glomerulus and 75% reabsorbed in the proximal tubule. Clearance is 20%–30% of glomerular filtration rate (GFR), typically 15–30 mL/min in adults with normal renal function	
*T*_1/2_, hours, range	18–36 h (adults with an average renal function) 40–50 h (impaired renal function)
CL, %, urine vs. feces	95% urine, less than 5% by feces
Steady‐state concentration	Steady‐state plasma concentrations are typically achieved within 4–7 days of consistent dosing. Therapeutic plasma concentrations for bipolar disorder are generally maintained between 0.6–1.2 mEq/L for acute mania and 0.4–1.0 mEq/L for maintenance therapy, monitored via trough levels (12 h post‐dose)
Factors affecting pharmacokinetics	*Renal function:* Impaired renal clearance (e.g., in chronic kidney disease) increases half‐life and risk of toxicity *Sodium balance:* Low sodium intake or dehydration increases lithium reabsorption in the proximal tubules, elevating plasma levels *Drug interactions:* Thiazide diuretics, nonsteroidal anti‐inflammatory drugs (NSAIDs), angiotensin II receptor blockers (ARBs), and angiotensin‐converting enzyme (ACE) inhibitors may reduce lithium clearance, increasing toxicity risk *Age:* Adults over 65 years of age exhibit reduced renal clearance, necessitating lower doses
Lithium Pharmacokinetics and therapeutic drug monitoring	Continuous monitoring of serum lithium levels is essential due to its narrow therapeutic range (toxic levels > 1.5 mEq/L) Renal function (e.g., serum creatinine, estimated glomerular filtration rate), thyroid function, and electrolyte balance should also be assessed every 3 months to mitigate long‐term adverse effects

Abbreviations: CL, clearance; *C*_max_, maximum plasma concentration; N/A, not applicable; *T*_1/2_, half‐life time; *T*_max_, time to maximum concentration; Tss, time to steady state; Vd/F, volume of distribution/bioavailability.

#### 2.2.1 Absorption

Lithium absorption occurs rapidly in the gastrointestinal tract, specifically in the small intestine. (3) Standard‐release formulations reach peak plasma concentrations within 1–2 h, whereas sustained‐release formulations do so in 4–5 h [38].

#### 2.2.2 Distribution

Lithium has a large volume of distribution (0.5–0.9 L/kg in adults), distributing throughout the body similarly to total body water. This indicates extensive tissue penetration and widespread distribution [39]. One of the most important characteristics of lithium is its slow cellular uptake, with some degree of accumulation observed in tissues such as bones and the thyroid gland, with an initial distribution half‐life of, on average, 5 h [40]. This drug does not significantly bind to plasma proteins and is unevenly distributed among different body compartments, including the brain, kidneys, and muscles [39]. Notably, lithium follows a bicompartmental pharmacokinetic profile, with rapid distribution in a central compartment (blood and highly perfused organs) followed by slower distribution in peripheral tissues (e.g., muscles and fat) [41]. For accurate therapeutic monitoring, serum lithium levels should be measured after the distribution phase is complete, generally 8–10 h after oral administration.

#### 2.2.3 Metabolism and Elimination

Lithium is not metabolized by the liver or any other organ; it undergoes no biotransformation in the human body, remaining unchanged. This distinguishes it from many other drugs that undergo hepatic metabolism [33]. Because lithium is not metabolized, its elimination does not depend on liver function. Instead, it is excreted unchanged, primarily through the kidneys, accounting for more than 95% of its clearance. Renal clearance ranges between 0.51 and 1.59 L/h and is closely related to kidney function [42].

Although lithium exhibits linear pharmacokinetics within the therapeutic range, its clearance can behave nonlinearly under clinical conditions. This is because, although it is eliminated primarily by glomerular filtration (a linear process), it also undergoes tubular reabsorption in the proximal nephron, competing with sodium. Factors such as sodium depletion, dehydration, or the concomitant use of diuretics, non‐steroidal anti‐inflammatory drugs (NSAIDs), or angiotensin‐converting enzyme (ACE) inhibitors can increase lithium reabsorption, reducing its elimination and causing disproportionate plasma accumulation. Therefore, although its basic kinetics are linear, its elimination can become nonlinear under common physiological or pharmacological influences. Additionally, lithium clearance may vary throughout the day, with lower rates observed at night compared to daytime [43]. The plasma elimination half‐life of lithium is 18–36 h and can vary depending on the patient's renal function [40]. During pregnancy, lithium clearance increases significantly (up to 63% in the third trimester) due to an increase in the glomerular filtration rate, further reinforcing its strong dependence on renal function [44].

## 3 Understanding Lithium's Potential as an Antidepressant Augmentation Agent

Lithium is a drug of choice for maintenance treatment of bipolar disorder; however, it has also shown efficacy in unipolar depressed patients as an augmentation therapy. Lithium augmentation was first reported in the 1980s to be effective in treating patients with depression who failed to respond to TCAs [45]. The effectiveness of lithium augmentation for depression was evaluated in the STAR*D (sequenced treatment alternatives to relieve depression) trial [46], where different antidepressant switching and augmentation therapies were examined [47]. The effectiveness of lithium augmentation was compared to triiodothyronine (T (3)) augmentation as a third‐step treatment for patients with MDD who had initially failed to remit to treatment with citalopram and subsequently failed to remit after a second antidepressant or augmentation strategy prior to randomization to lithium (up to 900 mg/day; *N* = 69) or T (3) (up to 50 μg/day; *N* = 73) for up to 2 weeks. In this trial, the remission rates were 15.9% with lithium augmentation and 24.7% with T (3) augmentation; the difference was not statistically significant. T (3) augmentation was concluded to be superior to lithium augmentation due to better tolerability of T (3) compared to lithium. However, this trial had limitations such as the lack of a placebo comparator, the open‐label administration of the augmentation therapies, and a study design that selects participants who have progressed through previous steps [46].

In a recent meta‐analysis, the efficacy and safety of SGA, esketamine, and lithium as antidepressant augmentation agents were compared. Randomized, placebo‐controlled trials were reviewed using meta‐analysis to compare the odds ratio (OR) to achieve response versus placebo [48]. They calculated the numbers needed to treat (NNT) and numbers needed to harm (NNH) when adding a SGA, esketamine, or lithium to antidepressants during major depressive episodes. The NNT for lithium was 5 (95% confidence interval (CI) 4–10) compared to SGAs with a NNT of 11 (95% CI: 9–15) and esketamine with a NNT of 7 (95% CI 4–10). Tolerability to lithium had a NNH of 9 (95% CI: 5–106); for SGAs, NNH was 5 (95% CI 4–6), and for esketamine, NNH was 5 (95% CI 4–6). Finally, the risk/benefit ratio was highest for lithium with an NNH/NNT of 1.80 (95% CI 1.25–10.60). The study demonstrates that SGA and esketamine are effective in combination with other antidepressants for acute major depressive episodes. However, lithium showed slightly higher efficacy and better tolerability compared to the other drugs [47].

Lithium is widely recognized for its ability to prevent relapse and recurrence of mood episodes, reduce aggression, impulsivity, and suicide risk, as well as reducing all‐cause mortality [49, 50]. Randomized clinical trials and meta‐analyses estimate a reduction in suicidal death of approximately 60%–70% with lithium and a reduction in all‐cause mortality of around 60% compared to placebo or other active treatments [16]. Lithium augmentation therapy is recommended in patients with TRD (see Table 3) and high risk of suicide; other indications include history of more than three major depressive episodes, psychomotor retardation, and weight loss. It also seems to be more effective in patients with a first‐degree relative with mood disorders [19].

**TABLE 3:** Selected treatment‐resistant depression and lithium studies.

Year and author	Type of article	Monotherapy	Combination	Neuromodulation (mECT)	Adverse reactions	Duration	Lithium dosage (augmentation therapy)	Lithium blood concentration	Diagnosis	Clinical outcome	References
Catalano et al. (2020)	Review of literature	NA	Lithium + SSRIs/TCAs	NA	NA	NA	600 mg/daily	NA	TRD	Lithium has both, a low unit cost and low NNT to TRD response (5) compared to aripiprazole, lithium, quetiapine, liothyronine, buspirone, brexipiprazole	[51]
Costi et al. (2019)	Double‐blind randomized controlled trial	NA	Lithium + Ketamine	NA	Nausea, headache, dry mouth, abdominal pain, constipation, pollakiuria	42 days	600–1200 mg/daily, 0.5 mg/kg infuse IV over 40 min	0.6–0.9 mEq/L	TRD	Participants randomized to lithium failed to show a prolonged reduction in MADRS score compared to those randomized to placebo	[52]
Osterland et al. (2023)	Multicenter Prospective Cohort Study	NA	Lithium + baseline treatment	NA	Dizziness, tremor, nausea	6 weeks	NS	0.5–0.9 mmol/L (≥ 65 year) 0.4–0.8 mmol/L (< 65 year)	Unipolar Depression	Both groups (≥ 65 year and < 65 year patients) showed a significant decline of eGFR under lithium augmentation	[53]
Bauer et al. (2003)	Systematic Review	NA	Lithium + TCA and tetracyclines	NA	NS	12–14 days	900–1200 mg/daily	NA	Bipolar disorder	Patients who respond to lithium augmentation should be maintained on lithium for a minimum of 12 months, or even longer. Lithium augmentation significantly increased prolactin response, compared with placebo pretreatment and antidepressant treatment alone	[6]
				NA		2 days	900 mg/daily		Bipolar disorder		
			Lithium + various TCA	NA		2 days	900 mg/daily		Unipolar disorder		
				NA		14 days	900–1200 mg/daily		Unipolar disorder		
				NA		1–21 days	250 mg/daily		Unipolar disorder		
						22–42	250 mg daily vs. 750 mg/daily				
			Lithium + various TCA and MAOI	NA		14 days	300–900 mg/daily		Unipolar disorder		
			Litium + various AD	NA		14 days	600–800 mg/daily		Bipolar disorder Unipolar disorder		
			Litium + SSRI and TCA	NA		42 days	400–800 mg/daily		Not reported		
			Litium + SSRI	NA		14 days	800 mg/daily		Bipolar disorder Unipolar disorder		
Woon et al. (2017)	Review	This review focuses on the use of lithium as monotherapy.	NA	NA	Hypothyroidism, hyperparathyroidism, and weight gain	NS	0.5–1.2 mmol/L	0.5–1.2 mmol/L (Therapeutic range)	Bipolar disorder	Neuroprotection, reduction in manic and depressive episodes, mood stabilization	[22]
Bauer et al. (2014)	Narrative Review	NA	Lithium used as augmentation therapy with antidepressants	NA	Polyuria, weight gain, tremor, nausea, hypothyroidism, renal effects	Variable (studies reviewed; suggested at least 1 year for maintenance)	Ranges from 300 mg/day to 1500 mg/day	0.5–0.8 mmol/L (recommended for augmentation)	Major Depressive Disorder (MDD) Treatment‐Resistant Depression Bipolar Disorder	Improved response and remission rates, significant efficacy in augmentation therapy	[45]
Rybakowski et al. (2020)	Narrative review	NA	NA	NA	Interstitial nephropaty, hypothyroidism	NA	Should not exceed 500 mg	0.6–0.8 mmol/L	Hypomania	Lower number of episodes, significantly reduced risk of rehospitalization, reduce suicide risk	[13]
								0.8–1.2 mmol/L	Mania		
			Lithium + MS					0.6–0.8 mmol/L	Rapid cycling		
								0.6–0.8 mmol/L	Prophylactic (long‐term prevention recurrences)		
			Lithium + SSRIs/TCAs/SNRI					0.6–0.8 mmol/L	Depression		
McKeown et al. (2022)	Qualitative	NA	Lithium (*n* = 32) vs. Quetiapine (*n* = 32) + existing antidepressant treatment	NA	Tremor, dry mouth, nausea	52 weeks	NA	NA	TRD	Patient experiences of lithium and quetiapine augmentation ‘Initial concerns’, ‘Experience of side effects’, ‘Perception of treatment efficacy’ and ‘Positive perception of treatment monitoring’. More concerns with lithium, but less side effects	[54]
Erci et al. (2023)	Narrative review	NA	Lithium + antidepressive agent	NA	tremor, GI, weight gain, hypothyroidism, hyperparathyroidism, nephrogenic diabetes insipidus, lithium nephropathy	NA	600–900 mg/day	0.5–0.8 mmol/L	TRD	Efficacy in prevention of relapses and recurrences of mood episodes and in TDR in patients who have responded to ECT. Reduced suicide risk	[19]
Terao et al. (2024)	Systematic review and meta‐analysis	NA	Combination therapy (ketamine, esketamine, aripiprazole, lithium)	NA	Ketamine: more acceptable, fewer adverse events. Esketamine and aripiprazole: less tolerable than placebo.	1 to 6 weeks depending on the study	Ketamine IV 0.5 mg/kg, Esketamine intranasal 28–84 mg, Aripiprazol variable, lithium variable	NS	Treatment‐resistant unipolar depression	IV ketamine more effective than esketamine and aripiprazole. Lithium not different from IV ketamine in efficacy, tolerability, and acceptance	[11]
Edwards et al. (2013)	Systematic review and economic evaluation	NA	Fluoxetine + Olanzapine, Fluoxetine + Lithium (dose not specified)	NA	Olanzapine: increased discontinuation rate. Lithium: lowered discontinuation rate compared to SSRI alone.	8 weeks of acute treatment and 10 months of maintenance	Fluoxetine + Olanzapine, Fluoxetine + Lithium (dose not specified)	NS	Treatment‐resistant unipolar depression	Olanzapine with SSRI showed superiority to SSRI alone. Lithium with SSRI showed positive trends but no significant differences	[5]
Undurraga et al. (2019)	Systematic review	Yes	Monotherapy and combination therapy (lithium alone or with antidepressants)	NA	General adverse effects of lithium not specifically detailed.	From 2 weeks to more than 12 months, depending on the study	NS	0.5–1.4 mEq/L in maintenance studies	Major unipolar depression, acute and long‐term treatment	Lithium effective in unipolar depression as an adjunct and for maintenance. Uncertain benefit in monotherapy	[55]
Patel et al. (2020)	Observational Study	NA	Combination of Lithium + ECT	(ECT combined with lithium) compared to either treatment alone	Higher risk of delirium and cognitive impairment (11.7 times more likely)	Acute inpatient treatment during hospitalization	NS	NS	Treatment‐resistant major depressive disorder, bipolar disorder (depressive and manic)	Higher risk of delirium in MDD (7.8%) than in BD (3.4%). Caution with combination is advised	[56]
Morlet et al. (2019)	Cross‐sectional Multicenter Study	NA	Monotherapy and combination with antidepressants	NA	Hypothyroidism, no increase in other medical comorbidities	Average of 12.5 years (SD = 11.6 years) in long‐term lithium users	59.1%: 723.8 mg sustained‐release formulation 15.9% ~ 546.4 mg (immediate‐release formulation) 25%: 487.5 mg lithium as an adjunct (sustained‐release formulation)	NS	Bipolar disorder and treatment‐resistant major depressive disorder	Reduction in depressive symptoms, lower benzodiazepine use, no impact on cognitive function	[57]
Bennabi et al. (2019)	Clinical Guidelines	NA	1st Intention Lithium, Quetiapine 2nd Intention Aripiprazole, Tri‐iodothyronine, Lamotrigine	ECT in monotherapy or in combination	NS	At least 6 months after remission; longer duration for recurrent cases	NS	0.5 to 0.8 mmol/L Measurement recommended	Treatment‐resistant depression	Recommendations for lithium treatment optimization, including plasma level monitoring	[10]
Rybakowski (2021)	Narrative Review	Used for prophylaxis of affective episodes in bipolar disorders and recurrent depression	Used to enhance antidepressant efficacy in TRD	NS	Nephrotoxicity and thyroid alterations; risk of tolerance with prolonged use.	More than 5 years	600–1800 mg daily (lithium carbonate)	0.6–1.2 mmol/L	Bipolar disorder and treatment‐resistant depression.	Prevention of affective relapses; reduction of suicide risk	[58]
Xiong et al. (2023)	Observational Genetic‐Based Study	NS	Used as an augmentation therapy for antidepressants in TRD	Used in severe cases of TRD resistant to antidepressants	No specific adverse events discussed.	No specific data on treatment duration.	NS	NS	Major depressive disorder (MDD)	Lithium shows genetic effectiveness in TRD patients; more favorable genetic profile for response	[59]
Vasquez et al. (2021)	Systematic Review and Meta‐Analysis	NS	Antidepressants + lithium, antidepressants + second‐generation antipsychotics (aripiprazole, olanzapine, fluoxetine, risperidone, ziprasidone)	NS	Tremor, dizziness, antipsychotic somnolence, akathisia.	Average of 3.4 weeks for lithium; 4 weeks for esketamine and 7 weeks for antipsychotics	NS	NS	Major depressive episodes (MDD).	Lithium is more effective as a combination therapy (NNT = 5) and has better tolerability compared to esketamine and antipsychotics	[48]
Cipriani et al. (2013)	Systematic Review and Meta‐Analysis	NA	Lithium + placebo or amitriptyline, carbamazepine, imipramine, lamotrigine, mianserin, maprotiline, nortriptyline, olanzapine, phenelzine, quetiapine, thyroid hormone	NA	NA	≥ 12 weeks	NS	NS	Unipolar depression, bipolar disorder	Lithium was associated with a reduced risk of suicide when compared with placebo, and also a reduce risk of deliberate self harm compared with carbamazepine, reduces risk of suicide and total deaths with both unipolar and bipolar depressive disorder	[16]
Baldessarini et al. (2006)	Meta‐Analysis	Lithium with or without other treatments	Lithium with or without other treatments	NS	NS	Any duration	Any dosages	NS	Major affective disorders	Lithium provides major reductions in the risk of completed and attempted suicides among BD and other major affective disorder patients during long‐term treatment with lithium	[15]

Abbreviations: AD, antidepressant; BD, bipolar disorder; eGFR, Estimated Glomerular Filtration Rate; MADRS, Montgomery–Åsberg Depression Rating Scale; MAOI, monoamine oxidase inhibitor; MDD, major depressive disorder; mECT, modified electroconvulsive therapy; MS, other mood stabilizer; NA, Non‐applicable; NNH, number needed to harm; NNT, number needed to treat; NS, not specified; SNRI, serotonin–norepinephrine reuptake inhibitorSSRI, selective serotonin reuptake inhibitor; TCA, tricyclic antidepressant; TRD, treatment resistant depression.

A systematic review of lithium to treat unipolar major depressive disorder documented six trials of lithium monotherapy and 12 trials of lithium augmentation to antidepressants for acute depression as well as 21 trials of lithium as monotherapy or adjunct to antidepressant treatment for long‐term prophylaxis [55]. Randomized trials included lithium as monotherapy or adjunctive with an antidepressant, compared to placebo or antidepressant monotherapy. Short‐term trials were defined as at least 1 week and up to 12 weeks of follow‐up, and long‐term trials as ≥ 12 weeks of follow‐up. Lithium monotherapy was not effective for the treatment of acute depressive episodes, where it was equivalent to placebo treatment. However, the efficacy of lithium as an augmentation agent to antidepressants favored lithium over placebo (OR 2.34 (95% CI 1.57–3.51); *p* < 0.0001). Interestingly, lithium monotherapy was superior to placebo, and lithium adjunctive therapy was superior to antidepressant monotherapy for long‐term prophylactic treatment (OR 2.80 (95% CI: 1.59–4.92); *p* < 0.0001).

One of the most recent meta‐analyses aimed to compare antidepressants (selective serotonin reuptake inhibitors (SSRIs), serotonin‐norepinephrine reuptake inhibitors (SNRIs), TCAs, monoamine oxidase inhibitors (MAOIs), norepinephrine‐dopamine reuptake inhibitors (DNRIs), agents targeting the glutamatergic system (e.g., ketamine and esketamine)), and augmentation agents (mood stabilizers, SGAs, thyroid hormones, anticonvulsants, and 5‐hydroxytryptamine (HT) partial agonist) on the basis of their efficacy, tolerability, and speed of symptom relief in TRD; the meta‐analyses included a total of 66 articles. Lithium was an augmentation agent in 16 articles (TCA: 9; SSRIs/SNRIs/NDRIs: 7). The study found that ketamine and esketamine, as well as lithium augmentation and combining antidepressants, were effective treatments for patients with TRD [60].

The use of lithium in combination with other augmentation agents, such as ketamine, has been studied in TRD. A randomized, double‐blind clinical trial conducted between 2013 and 2016 compared ketamine infusions plus lithium versus ketamine infusions with a placebo in patients with treatment‐resistant unipolar depression. Following an initial session of ketamine infusion, patients were randomized to receive lithium or placebo, as well as three additional sessions of ketamine. The results indicated no significant difference in depression severity between the two groups, as measured by the Montgomery–Åsberg Depression Rating Scale (MADRS), suggesting no additional improvement with the addition of lithium [52].

The presence of some adverse effects such as weight gain, tremor, thyroid dysfunction, and gastrointestinal dysfunction could influence adherence to lithium therapy. In a qualitative assessment aimed at evaluating opinions of lithium and quetiapine augmentation in patients with TRD, four main themes were generated as follows: initial concerns, experience of side effects, perception of treatment efficacy, and positive perception of treatment monitoring. Participants indicated a positive experience of lithium and quetiapine augmentation, although some patients held beliefs that suggested greater apprehension towards lithium than quetiapine [54]. This suggests that appropriate education should be provided to every patient and their families before initiating lithium therapy, addressing questions, doubts, and the possible fears patients might have.

The combination of neuromodulation therapies, such as electroconvulsive therapy (ECT) with lithium, has generated conflicting data. Although this approach appears to be more effective than either lithium or ECT alone, evidence suggests an increased risk of delirium and neurocognitive side effects. An observational study of 64,728 patients, examining the association between these adverse effects and the combined use of ECT and lithium, compared to ECT alone, found a higher risk of acute delirium with ECT and lithium in patients with major depressive disorder (7.8%) compared to those with bipolar disorder (3.4%). Cognitive impairment was also higher for all patients pooled who received a combination of lithium and ECT (2.4%) versus ECT only (0.5%). Therefore, clinicians should exercise caution when using this combination. However, available data remain limited [56].

On the other hand, following a successful course of ECT for depression, continuation ECT combined with lithium has been shown to reduce the risk of relapse. A systematic review and meta‐analysis that included 14 studies and 9748 patients receiving continuation ECT (*N* = 1571 with lithium and *N* = 8177 without lithium), with follow‐up durations ranging from 15 to 58 weeks, found that the group treated with lithium had significantly lower relapse rates, with a weighted OR of 0.53 and a NNT of 7 [61].

Although lithium is used more cautiously in geriatric patients because of the differences in efficacy and tolerability among older patients compared to young patients [57], it is important to know how to use it and what care needs to be taken when it is used in older patients. Adults ≥ 65 years of age require lower doses of lithium to achieve similar plasma concentrations because of age‐related changes, such as a reduced glomerular filtration rate. A prospective multicenter cohort study investigated the estimated glomerular filtration (eGFR) rate changes and number of acute kidney injuries (AKI) following lithium augmentation therapy in adults ≥ 65 years compared with patients < 65 years of age. The study showed that two variables, age ≥ 65 years and serum lithium levels, had a negative effect on eGFR. Two AKIs were observed in the ≥ 65‐years group when serum lithium exceeded the therapeutic range of > 0.8 mmol/L. [53] The recommended target serum lithium levels should be < 0.6 mmol/L in this population.

## 4 Key Factors Influencing Lithium Treatment Response

About half of individuals with depression show an inadequate response to lithium augmentation, even though it has demonstrated greater efficacy compared to placebo [6]. Several biological variables include a higher cortisol–adrenocorticotropic hormone (ACTH) ratio in the dexamethasone suppression‐corticotropin‐releasing hormone stimulation test (DEX‐CRH) in poorer responders, in which, after stimulation of the hypothalamus–pituitary–adrenocortical (HPA) system by ACTH, cortisol and ACTH are raised due to a sensitivity to the adrenal cortex. Evidence demonstrates a higher ratio of non‐response in patients with a more chronic depression course, given that one of the biological changes associated with chronic illness is the enlargement of the adrenal gland with an increased sensitivity to ACTH. Studies in rats showed an increase in the neuroprotective protein B‐cell lymphoma 2 (bcl‐2) in the frontal cortex and hippocampus and an increased expression of the major protein kinase C (PKC) substrate, myristoylated alanine‐rich C‐kinase substrate (MARCKS) [6, 58].

Patients who present with severe depressive symptomatology, weight loss, psychomotor retardation, a history of more than three major depressive episodes, and a first‐degree relative with mood disorders may have shown better outcomes from lithium therapy [45].

In 2009, the International Consortium on Lithium Genetics (ConLiGen) was established with the goal of conducting the first genome‐wide association study (GWAS) on lithium response in 2563 individuals with bipolar disorder. This landmark study identified genetic markers associated with long noncoding RNAs. More recent research has demonstrated that polygenic scores for certain psychiatric disorders, such as MDD and schizophrenia, are linked to poor lithium response [58]. These findings highlight lithium's limited effectiveness in patients with a genetic predisposition to psychotic symptoms.

Ying Xiong et al. conducted a study to explore the relationship between polygenic risk scores (PRS) and treatment resistance in MDD. They analyzed 4500 individuals with MDD, defining two phenotypes: TRD and non‐resistant depression (non‐TRD). PRS were generated for both antidepressant and lithium responses. Although no significant differences were found in PRS for antidepressant responses between the two groups, TRD cases showed significantly higher PRS for lithium response compared with non‐TRD cases [59]. These results suggest a genetic predisposition influencing lithium sensitivity in TRD.

## 5 Advantages of Lithium in the Clinical Context

Edwards et al. conducted a systematic review and economic evaluation of lithium and SGA in the management of TRD. In this study, costs generally included laboratory monitoring—such as lithium plasma level determinations and renal and thyroid function tests—along with other clinical assessments and related visits. They identified four economic evaluations in the management of TRD and five studies that reported utility values for different levels of depression severity and treatment response. The systematic review showed that the annual cost per patient treated with SSRI + lithium was estimated to be €4739 compared with €5644 for those treated with SSRI + SGA [5], supporting lithium as an option for long‐term treatment for either bipolar disorder or TRD.

These results align with a review by Catalano et al. in 2020, which examined the cost‐effectiveness of lithium combined with thyroid hormones, aripiprazole, brexpiprazole, quetiapine, and buspirone. The study concluded that lithium offered both a low unit cost ($6.42) and a low NNT (5) compared to the other agents. In contrast, although quetiapine appears to be a low‐cost option, its higher NNT (10) results in a greater cost per patient to achieve remission ($140.40 quetiapine vs. $96.30 lithium) [51].

Evidence supports the use of lithium in the prevention of hospitalization for patients with mood disorders. In a cohort study which aimed to assess the risk of rehospitalization of 123,712 patients with severe recurrent depression between 1996 and 2012, lithium was associated with a reduced risk of rehospitalization. In addition, treatment with lithium plays a crucial role in reducing mortality, particularly by preventing suicide [13]. A meta‐analysis conducted by Baldessarini et al. included 45 studies with data on suicides that occurred during an average of 1.5 years of lithium treatment as well as 34 studies reporting suicides among individuals who did not receive lithium. The results showed that the risk of suicide was five times lower in patients treated with lithium compared to those who received other treatments [15].

In recent years, the neuroprotective effect of lithium has been studied in mood and neurocognitive disorders [13]. Retrospective studies have demonstrated that patients on long‐term lithium therapy were much less likely to develop neurological disorders such as dementia, seizures, or amyotrophic lateral sclerosis. This has been associated with lithium's role in modulating autophagy as well as its ability to mitigate neuroinflammation and to preserve and even increase telomere length [62, 63].

To maximize safety, before starting treatment with lithium, every patient should have a baseline thyroid hormone evaluation including thyroid‐stimulating hormone (TSH). Kidney function tests also should be measured, including sodium levels, given their direct relationship with lithium and electrocardiogram (EKG) if patients have cardiovascular risk factors or are ≥ 40 years of age. An empirical starting dose of 300 mg twice daily is frequently used for lithium augmentation in clinical practice; however, individualized dose calculations based on pharmacokinetic principles may allow faster attainment of therapeutic concentrations, optimizing both clinical outcomes and resource use. Because of the toxicity risk with a prolonged treatment duration and high doses, lithium serum levels should be monitored every 3–6 months, unless another medication that can alter lithium levels is initiated, in which case lithium serum levels should be monitored more frequently. More frequent monitoring is also recommended in cases of dose adjustments, changes in renal function, or when clinical signs of toxicity appear. Target lithium serum levels are 0.5–0.8 mEq/L [19].

## 6 Conclusions

The evidence reviewed in this article suggests that lithium augmentation is a suitable option for the treatment of TRD, as it helps enhance the effects of other antidepressant agents and offers advantages such as reducing hospitalizations and lowering mortality by reducing suicide. However, strict monitoring is required to reduce adverse reactions and complications from long‐term use. Lithium is a cost‐effective drug that may have a role in the management of TRD. Nevertheless, many studies evaluating lithium augmentation in TRD were conducted in the past decades using older antidepressants. More updated studies that include newer antidepressants are needed to evaluate lithium augmentation and its nuances in TRD.

## Author Contributions

**Angela Acero‐González:** writing – original draft, writing – review and editing. **Yahira Guzman:** supervision, writing – original draft, writing – review and editing. **Nadia Juliana Proaños:** writing – original draft, writing – review and editing. **Rosa‐Helena Bustos:** writing – original draft, writing – review and editing. **María Aconcha:** writing – original draft, writing – review and editing. **Ivan Guerrero:** writing – original draft, writing – review and editing. **Laura Alejandra Martinez:** writing – original draft, writing – review and editing. **Michael Berk:** writing – original draft, writing – review and editing. **Seetal Dodd:** writing – original draft, writing – review and editing.

## Conflicts of Interest

M.B. is supported by a NHMRC senior principal research fellowship and leadership 3 investigator grant (1156072 and 2017131), has received grant funding from Wellcome Trust, MRFF, Victorian Government Department of Jobs, Precincts and Regions, Cooper University USA, Janssen Lundbeckfonden Copenhagen, St. Biopharma, Psychscene.com, WFSBP, NeuroSAS, CINP, Shanghai Mental Health Center, Penn State College of Medicine, Precision Psych Fondamental, ISBD, Milken Baszucki Brain Research Fund, Stanley Medical Research Institute, Danmarks Frie Forskningsfond Psykiatrisk Center Kovenhavn, Patient‐Centered Outcomes Research Institute (PCORI), Australian Eating Disorders Research and Translation Centre AEDRTC, USA Department of Defense Office of the Congressionally Directed Medical Research Programs (CDMRP), Equity Trustees Limited, and has provided lectures for Global Congress of Biological Psychiatry India, Otsuka CNS, RANZCP New Zealand, Eisai Australia, Sandoz, Allori, Lundbeck, World Congress of Psychiatry, African College of Neuropsychopharmacology, SVI Inaugural Health Matters Webinar Series, Argeninte Association of Psychiatrists Congress of Psychiatry and Mental Health, Global Bipolar Cohort (GBC). S.D. has received grant support from the Stanley Medical Research Institute, NHMRC, Beyond Blue, ARHRF, Simons Foundation, Geelong Medical Research Foundation, Harry Windsor Foundation, Fondation FondaMental, Eli Lilly, Glaxo SmithKline, Organon, Mayne Pharma and Servier, speaker's fees from Eli Lilly, advisory board fees from Eli Lilly and Novartis, and conference travel support from Servier. All other authors declare no conflicts of interest.

## Data Availability

Data available on request from the authors.

## References

[1] A. J. Ferrari , D. F. Santomauro , A. Aali , et al., “Global Incidence, Prevalence, Years Lived With Disability (YLDs), Disability‐Adjusted Life‐Years (DALYs), and Healthy Life Expectancy (HALE) for 371 Diseases and Injuries in 204 Countries and Territories and 811 Subnational Locations, 1990–2021: A Systematic Analysis for the Global Burden of Disease Study 2021,” Lancet 403, no. 10440 (2024): 2133–2161. 38642570 10.1016/S0140-6736(24)00757-8 PMC11122111

[2] World Health Organization , “World Mental Health Report: Transforming Mental Health for All,” accessed January 13, 2025, https://www.who.int/publications/i/item/9789240049338.

[3] Association AP , Diagnostic and Statistical Manual of Mental Disorders, vol. 21, 5th ed. (Association AP, 2022), 591–643.

[4] R. S. McIntyre , M. Alsuwaidan , B. T. Baune , et al., “Treatment‐Resistant Depression: Definition, Prevalence, Detection, Management, and Investigational Interventions,” World Psychiatry: Official Journal of the World Psychiatric Association 22, no. 3 (2023): 394–412. 10.1002/wps.21120 PMC10503923 37713549

[5] S. J. Edwards , V. Hamilton , L. Nherera , and N. Trevor , “Lithium or an Atypical Antipsychotic Drug in the Management of Treatment‐Resistant Depression: A Systematic Review and Economic Evaluation,” Health Technology Assessment 17, no. 54 (2013): 1–190. 10.3310/hta17540 PMC4781298 24284258

[6] M. Bauer , M. Adli , C. Baethge , et al., “Lithium Augmentation Therapy in Refractory Depression: Clinical Evidence and Neurobiological Mechanisms,” Canadian Journal of Psychiatry. Revue Canadienne de Psychiatrie 48, no. 7 (2003): 440–448. 12971013 10.1177/070674370304800703

[7] G. Li , D. Fife , G. Wang , et al., “All‐Cause Mortality in Patients With Treatment‐Resistant Depression: A Cohort Study in the US Population,” Annals of General Psychiatry 18 (2019): 23. 31583010 10.1186/s12991-019-0248-0 PMC6771113

[8] R. D. Sousa , M. Gouveia , C. Nunes da Silva , et al., “Treatment‐Resistant Depression and Major Depression With Suicide Risk‐The Cost of Illness and Burden of Disease,” Frontiers in Public Health 10 (2022): 898491. 36033799 10.3389/fpubh.2022.898491 PMC9402971

[9] B. N. Gaynes , L. Lux , G. Gartlehner , et al., “Defining Treatment‐Resistant Depression,” Depression and Anxiety 37, no. 2 (2020): 134–145. 31638723 10.1002/da.22968

[10] D. Bennabi , T. Charpeaud , A. Yrondi , et al., “Clinical Guidelines for the Management of Treatment‐Resistant Depression: French Recommendations From Experts, the French Association for Biological Psychiatry and Neuropsychopharmacology and the Fondation FondaMental,” BMC Psychiatry 19, no. 1 (2019): 262. 31455302 10.1186/s12888-019-2237-x PMC6712810

[11] I. Terao , T. Tsuge , K. Endo , and W. Kodama , “Comparative Efficacy, Tolerability and Acceptability of Intravenous Racemic Ketamine With Intranasal Esketamine, Aripiprazole and Lithium as Augmentative Treatments for Treatment‐Resistant Unipolar Depression: A Systematic Review and Network Meta‐Analysis,” Journal of Affective Disorders 346 (2024): 49–56. 37949235 10.1016/j.jad.2023.11.023

[12] L. N. Yatham , S. H. Kennedy , S. V. Parikh , et al., “Canadian Network for Mood and Anxiety Treatments (CANMAT) and International Society for Bipolar Disorders (ISBD) 2018 Guidelines for the Management of Patients With Bipolar Disorder,” Bipolar Disorders 20, no. 2 (2018): 97–170. 29536616 10.1111/bdi.12609 PMC5947163

[13] J. K. Rybakowski , “Lithium—Past, Present, Future,” International Journal of Psychiatry in Clinical Practice 24, no. 4 (2020): 330–340. 33169645 10.1080/13651501.2020.1775855

[14] L. V. Kessing , M. Bauer , W. A. Nolen , E. Severus , G. M. Goodwin , and J. Geddes , “Effectiveness of Maintenance Therapy of Lithium vs Other Mood Stabilizers in Monotherapy and in Combinations: A Systematic Review of Evidence From Observational Studies,” Bipolar Disorders 20, no. 5 (2018): 419–431. 10.1111/bdi.12623 29441712

[15] R. J. Baldessarini , L. Tondo , P. Davis , M. Pompili , F. K. Goodwin , and J. Hennen , “Decreased Risk of Suicides and Attempts During Long‐Term Lithium Treatment: A Meta‐Analytic Review,” Bipolar Disorders 8, no. 5 Pt 2 (2006): 625–639. 17042835 10.1111/j.1399-5618.2006.00344.x

[16] A. Cipriani , K. Hawton , S. Stockton , and J. R. Geddes , “Lithium in the Prevention of Suicide in Mood Disorders: Updated Systematic Review and Meta‐Analysis,” BMJ 346 (2013): f3646. 23814104 10.1136/bmj.f3646

[17] J. F. Cade , “Lithium Salts in the Treatment of Psychotic Excitement. 1949,” Bulletin of the World Health Organization 78, no. 4 (2000): 518–520. 10885180 PMC2560740

[18] A. Serretti , A. Drago , and D. De Ronchi , “Lithium Pharmacodynamics and Pharmacogenetics: Focus on Inositol Mono Phosphatase (IMPase), Inositol Poliphosphatase (IPPase) and Glycogen Sinthase Kinase 3 Beta (GSK‐3 Beta),” Current Medicinal Chemistry 16, no. 15 (2009): 1917–1948. 19442155 10.2174/092986709788186101

[19] M. Ercis , A. Ozerdem , and B. Singh , “When and How to Use Lithium Augmentation for Treating Major Depressive Disorder,” Journal of Clinical Psychiatry 84, no. 2 (2023): 23ac14813. 10.4088/JCP.23ac14813 36883886

[20] A. G. Pacholko and L. K. Bekar , “Lithium Orotate: A Superior Option for Lithium Therapy?,” Brain and Behavior: A Cognitive Neuroscience Perspective 11, no. 8 (2021): e2262. 10.1002/brb3.2262 PMC8413749 34196467

[21] D. Chatterjee and J. M. Beaulieu , “Inhibition of Glycogen Synthase Kinase 3 by Lithium, a Mechanism in Search of Specificity,” Frontiers in Molecular Neuroscience 15 (2022): 1028963. 36504683 10.3389/fnmol.2022.1028963 PMC9731798

[22] E. Won and Y. K. Kim , “An Oldie but Goodie: Lithium in the Treatment of Bipolar Disorder Through Neuroprotective and Neurotrophic Mechanisms,” International Journal of Molecular Sciences 18, no. 12 (2017): 2679. 29232923 10.3390/ijms18122679 PMC5751281

[23] D. Liu , Q. Q. Tang , D. Wang , et al., “Mesocortical BDNF Signaling Mediates Antidepressive‐Like Effects of Lithium,” Neuropsychopharmacology: Official Publication of the American College of Neuropsychopharmacology 45, no. 9 (2020): 1557–1566. 32428928 10.1038/s41386-020-0713-0 PMC7360776

[24] T. Dudev , K. Mazmanian , W.‐H. Weng , C. Grauffel , and C. Lim , “Free and Bound Therapeutic Lithium in Brain Signaling,” Accounts of Chemical Research 52, no. 10 (2019): 2960–2970. 31556294 10.1021/acs.accounts.9b00389

[25] F. Ghanaatfar , A. Ghanaatfar , P. Isapour , et al., “Is Lithium Neuroprotective? An Updated Mechanistic Illustrated Review,” Fundamental & Clinical Pharmacology 37, no. 1 (2023): 4–30. 35996185 10.1111/fcp.12826

[26] Ł. P. Szałach , K. A. Lisowska , W. J. Cubała , M. Barbuti , and G. Perugi , “The Immunomodulatory Effect of Lithium as a Mechanism of Action in Bipolar Disorder,” Frontiers in Neuroscience 17 (2023): 1213766. 37662097 10.3389/fnins.2023.1213766 PMC10469704

[27] M. J. Berridge and R. F. Irvine , “Inositol Phosphates and Cell Signalling,” Nature 341, no. 6239 (1989): 197–205. 2550825 10.1038/341197a0

[28] A. Cuadrado , E. Cazalla , A. Bach , et al., “Health Position Paper and Redox Perspectives—Bench to Bedside Transition for Pharmacological Regulation of NRF2 in Noncommunicable Diseases,” Redox Biology 81 (2025): 103569. 40059038 10.1016/j.redox.2025.103569 PMC11970334

[29] D. C. Rubinsztein , P. Codogno , and B. Levine , “Autophagy Modulation as a Potential Therapeutic Target for Diverse Diseases,” Nature Reviews Drug Discovery 11, no. 9 (2012): 709–730. 22935804 10.1038/nrd3802 PMC3518431

[30] A. Bortolozzi , G. Fico , M. Berk , et al., “New Advances in the Pharmacology and Toxicology of Lithium: A Neurobiologically Oriented Overview,” Pharmacological Reviews 76, no. 3 (2024): 323–357. 38697859 10.1124/pharmrev.120.000007 PMC11068842

[31] A. J. Harwood , “Lithium and Bipolar Mood Disorder: The Inositol‐Depletion Hypothesis Revisited,” Molecular Psychiatry 10, no. 1 (2005): 117–126. 15558078 10.1038/sj.mp.4001618

[32] J. Methaneethorn , “Population Pharmacokinetic Analyses of Lithium: A Systematic Review,” European Journal of Drug Metabolism and Pharmacokinetics 43, no. 1 (2018): 25–34. 28555320 10.1007/s13318-017-0421-2

[33] J. Wen , D. Sawmiller , B. Wheeldon , and J. Tan , “A Review for Lithium: Pharmacokinetics, Drug Design, and Toxicity,” CNS & Neurological Disorders Drug Targets 18, no. 10 (2019): 769–778. 31724518 10.2174/1871527318666191114095249

[34] Drugbank , “Lithium Carbonate,” accessed February 15, 2025, https://go.drugbank.com/drugs/DB14509.

[35] K. M. Brown and D. K. Tracy , “Lithium: The Pharmacodynamic Actions of the Amazing Ion,” Therapeutic Advances in Psychopharmacology 3, no. 3 (2013): 163–176. 24167688 10.1177/2045125312471963 PMC3805456

[36] C. Couffignal , L. Chevillard , S. El Balkhi , S. Cisternino , and X. Declèves , “The Pharmacokinetics of Lithium,” in The Science and Practice of Lithium Therapy, ed. G. S. Malhi , M. Masson , and F. Bellivier (Springer International Publishing, 2017), 25–53.

[37] K. Chokhawala , S. Lee , and A. Saadabadi , “Lithium,” in StatPearls (StatPearls Publishing LLC, 2025). 30085604

[38] E. M. Grandjean and J. M. Aubry , “Lithium: Updated Human Knowledge Using an Evidence‐Based Approach. Part II: Clinical Pharmacology and Therapeutic Monitoring,” CNS Drugs 23, no. 4 (2009): 331–349. 19374461 10.2165/00023210-200923040-00005

[39] P. A. Luisier , P. Schulz , and P. Dick , “The Pharmacokinetics of Lithium in Normal Humans: Expected and Unexpected Observations in View of Basic Kinetic Principles,” Pharmacopsychiatry 20, no. 5 (1987): 232–234. 3118402 10.1055/s-2007-1017112

[40] M. E. Ward , M. N. Musa , and L. Bailey , “Clinical Pharmacokinetics of Lithium,” Journal of Clinical Pharmacology 34, no. 4 (1994): 280–285. 8006194 10.1002/j.1552-4604.1994.tb01994.x

[41] L. A. Bauer , “Chapter 17. Lithium,” in Applied Clinical Pharmacokinetics, 2e, ed. L. A. Bauer (McGraw‐Hill Companies, 2008).

[42] J. Yuan , B. Zhang , Y. Xu , et al., “Population Pharmacokinetics of Lithium in Young Pediatric Patients With Intellectual Disability,” Frontiers in Pharmacology 12 (2021): 650298. 33935755 10.3389/fphar.2021.650298 PMC8082156

[43] Z. B. Jin , Z. Wu , Y. F. Cui , et al., “Population Pharmacokinetics and Dosing Regimen of Lithium in Chinese Patients With Bipolar Disorder,” Frontiers in Pharmacology 13 (2022): 913935. 35860024 10.3389/fphar.2022.913935 PMC9289112

[44] C. T. Clark , R. L. Newmark , K. L. Wisner , C. Stika , and M. J. Avram , “Lithium Pharmacokinetics in the Perinatal Patient With Bipolar Disorder,” Journal of Clinical Pharmacology 62, no. 11 (2022): 1385–1392. 35620848 10.1002/jcph.2089 PMC9796861

[45] M. Bauer , M. Adli , R. Ricken , E. Severus , and M. Pilhatsch , “Role of Lithium Augmentation in the Management of Major Depressive Disorder,” CNS Drugs 28, no. 4 (2014): 331–342. 24590663 10.1007/s40263-014-0152-8

[46] A. A. Nierenberg , M. Fava , M. H. Trivedi , et al., “A Comparison of Lithium and T(3) Augmentation Following Two Failed Medication Treatments for Depression: A STAR*D Report,” American Journal of Psychiatry 163, no. 9 (2006): 1519–1530. 16946176 10.1176/ajp.2006.163.9.1519

[47] A. J. Rush , M. Fava , S. R. Wisniewski , et al., “Sequenced Treatment Alternatives to Relieve Depression (STAR*D): Rationale and Design,” Controlled Clinical Trials 25, no. 1 (2004): 119–142. 15061154 10.1016/s0197-2456(03)00112-0

[48] G. H. Vázquez , A. Bahji , J. Undurraga , L. Tondo , and R. J. Baldessarini , “Efficacy and Tolerability of Combination Treatments for Major Depression: Antidepressants Plus Second‐Generation Antipsychotics vs. Esketamine vs. Lithium,” Journal of Psychopharmacology 35, no. 8 (2021): 890–900. 34238049 10.1177/02698811211013579 PMC8358538

[49] G. Anmella , G. Fico , M. Lotfaliany , et al., “Risk of Cancer in Bipolar Disorder and the Potential Role of Lithium: International Collaborative Systematic Review and Meta‐Analyses,” Neuroscience and Biobehavioral Reviews 126 (2021): 529–541. 33831461 10.1016/j.neubiorev.2021.03.034

[50] K. Ponzer , V. Millischer , M. Schalling , M. Gissler , C. Lavebratt , and L. Backlund , “Lithium and Risk of Cardiovascular Disease, Dementia and Venous Thromboembolism,” Bipolar Disorders 25, no. 5 (2023): 391–401. 36651280 10.1111/bdi.13300

[51] G. Catalano , R. A. Robeel , G. A. Cheney , et al., “Antidepressant Augmentation: A Review of the Literature and a Review of the Pharmacoeconomic Considerations,” Journal of Clinical Psychopharmacology 40, no. 4 (2020): 396–400. 32639292 10.1097/JCP.0000000000001236

[52] S. Costi , L. Soleimani , A. Glasgow , et al., “Lithium Continuation Therapy Following Ketamine in Patients With Treatment Resistant Unipolar Depression: A Randomized Controlled Trial,” Neuropsychopharmacology: Official Publication of the American College of Neuropsychopharmacology 44, no. 10 (2019): 1812–1819. 30858518 10.1038/s41386-019-0365-0 PMC6784998

[53] S. L. Osterland , M. Adli , T. Saritas , et al., “Acute Effects of Lithium Augmentation on the Kidney in Geriatric Compared With Non‐Geriatric Patients With Treatment‐Resistant Depression,” Acta Psychiatrica Scandinavica 147, no. 3 (2023): 267–275. 36585782 10.1111/acps.13531

[54] L. McKeown , R. W. Taylor , E. Day , et al., “Patient Perspectives of Lithium and Quetiapine Augmentation Treatment in Treatment‐Resistant Depression: A Qualitative Assessment,” Journal of Psychopharmacology 36, no. 5 (2022): 557–565. 35475375 10.1177/02698811221089042 PMC9112618

[55] J. Undurraga , K. Sim , L. Tondo , et al., “Lithium Treatment for Unipolar Major Depressive Disorder: Systematic Review,” Journal of Psychopharmacology 33, no. 2 (2019): 167–176. 30698058 10.1177/0269881118822161

[56] R. S. Patel , A. Bachu , and N. A. Youssef , “Combination of Lithium and Electroconvulsive Therapy (ECT) is Associated With Higher Odds of Delirium and Cognitive Problems in a Large National Sample Across the United States,” Brain Stimulation 13, no. 1 (2020): 15–19. 31492631 10.1016/j.brs.2019.08.012

[57] E. Morlet , J. F. Costemale‐Lacoste , E. Poulet , K. McMahon , N. Hoertel , and F. Limosin , “Psychiatric and Physical Outcomes of Long‐Term Use of Lithium in Older Adults With Bipolar Disorder and Major Depressive Disorder: A Cross‐Sectional Multicenter Study,” Journal of Affective Disorders 259 (2019): 210–217. 31446382 10.1016/j.jad.2019.08.056

[58] J. K. Rybakowski , “Lithium Treatment in the Era of Personalized Medicine,” Drug Development Research 82, no. 5 (2021): 621–627. 32207857 10.1002/ddr.21660

[59] Y. Xiong , R. Karlsson , J. Song , et al., “Polygenic Risk Scores of Lithium Response and Treatment Resistance in Major Depressive Disorder,” Translational Psychiatry 13, no. 1 (2023): 301. 37770441 10.1038/s41398-023-02602-3 PMC10539379

[60] V. L. Ruberto , M. K. Jha , and J. W. Murrough , “Pharmacological Treatments for Patients With Treatment‐Resistant Depression,” Pharmaceuticals 13, no. 6 (2020): 116. 32512768 10.3390/ph13060116 PMC7345023

[61] S. Lambrichts , J. Detraux , K. Vansteelandt , et al., “Does Lithium Prevent Relapse Following Successful Electroconvulsive Therapy for Major Depression? A Systematic Review and Meta‐Analysis,” Acta Psychiatrica Scandinavica 143, no. 4 (2021): 294–306. 33506961 10.1111/acps.13277

[62] V. J. R. De‐Paula , M. Radanovic , and O. V. Forlenza , “Lithium and Neuroprotection: A Review of Molecular Targets and Biological Effects at Subtherapeutic Concentrations in Preclinical Models of Alzheimer's Disease,” International Journal of Bipolar Disorders 13, no. 1 (2025): 16. 40348943 10.1186/s40345-025-00386-7 PMC12065699

[63] S. Puglisi‐Allegra , G. Lazzeri , C. L. Busceti , F. S. Giorgi , F. Biagioni , and F. Fornai , “Lithium Engages Autophagy for Neuroprotection and Neuroplasticity: Translational Evidence for Therapy,” Neuroscience and Biobehavioral Reviews 148 (2023): 105148. 36996994 10.1016/j.neubiorev.2023.105148

